# Disseminated *M. bovis* Infection and Vertebral Osteomyelitis following Immunotherapy for Bladder Cancer

**DOI:** 10.1155/2020/6676163

**Published:** 2020-12-28

**Authors:** Harpreet S. Gill, Suhas P. Dasari, Sarbagya Pandit, Thomas E. Ritter, Sushma B. Raju, Alexander R. Chartier, Pinky Jha

**Affiliations:** Department of Internal Medicine, Medical College of Wisconsin, Wauwatosa, Wisconsin, USA

## Abstract

The use of BCG in immunotherapy for bladder cancer has been in practice for over 40 years. However, uncommon, serious complications can occur with the therapy. Here, we present a case of vertebral osteomyelitis secondary to dissemination of BCG following immunotherapy, an exceedingly rare presentation of an already rare complication.

## 1. Introduction

Bacillus Calmette–Guerin (BCG) *Mycobacterium bovis* (*M*. *bovis)* is used in immunotherapy for superficial bladder cancer and has been shown to delay tumor progression, decrease need for subsequent cystectomy, and improve survival [1]. Although generally well tolerated, local and systemic infections manifesting as malaise, bladder irritation, and fever can occur. While the method of metastasis is debated, lymphatic and hematogenous spread through disrupted uroepithelial cells and seeding in the spine is postulated [2, 3]. Here, we present a case of vertebral osteomyelitis of the lumbar spine secondary to systemic dissemination.

## 2. Case Presentation

We present the case of a 76-year-old male with a history of benign prostatic hyperplasia and bladder cancer diagnosed in 2018. Following a transurethral resection of bladder tumor (TURBT) procedure, he received intravesical BCG. In May 2019, he presented with severe back pain and radiculopathy. A lumbar MRI study showed multilevel degeneration, severe canal stenosis, and morphological changes suggestive of infection for which he received L1–L5 laminectomies and foraminotomies. Three weeks later, he presented with recurrent back and hip pain. A repeat MRI study revealed progressive degeneration and a paraspinal mass. A biopsy showed granulomatous inflammation which stained negative for AFP, malignancy, and neoplasm. In addition, normal blood cell counts were not suggestive of infection. Two weeks later, he returned with night sweats, fever, and severe pain in his back and right lower extremity; imaging revealed nonenhancing psoas abscesses. Pathology from the spinal biopsy demonstrated chronic, necrotizing osteomyelitis/discitis. Biopsy of the psoas abscess showed fragments of the skeletal muscle with severe inflammation and necrotizing granuloma. Labs obtained at that time showed WBC: 8.6 K/uL (3.5–11.0 K/uL), hemoglobin: 11.1 g/dL (13.0–17.0 g/dL), platelet count: 231 K/uL (130–450 K/uL), BUN: 24 mg/dL (6–23 mg/dL), creatinine: 1.19 mg/dL (0.70–1.30 mg/dL), bicarbonate: 24 mmol/L (22–29 mmol/L), ESR: 72 mm/hr (<30 mm/hr), and CRP: 3.30 mg/L (1–3 mg/L).

Cultures and urinalysis for multiple infectious agents were negative, but given his recent intravesical BCG treatment in August 2019, *Mycobacterium* infection was highly suspected, and the patient was started on empiric treatment with rifampin, isoniazid, and ethambutol.

His back pain, fever, and night sweats improved initially, but after 3-4 weeks, the fever and back pain returned. Repeat labs showed WBC: 7.3 K/uL (3.5–11.0 K/uL), ALT: 81 U/L (8–66 U/L), AST: 75 U/L (13–44 U/L), ALP: 317 U/L (40–129 U/L), bilirubin: 0.4 mg/dL (0.2–1.3 mg/dL), ESR: 79 mm/hr (<30 mm/hr), and CRP: 2 mg/L (1–3 mg/L).

He was started on vancomycin and cefepime in addition to the anti-mycobacterials for wider bacterial targeting. At this time, culture from the prior spinal biopsy returned positive for the *Mycobacterium tuberculosis* complex, and PCR was positive for *M. bovis* with pansensitivity. Broad-spectrum antibiotics were discontinued, and a steroid taper was started. In the following days, the patient's fever and back pain slowly improved.

In December 2019, he underwent spinal stabilization surgery and completed ethambutol therapy. His symptoms have since resolved, and he continues to follow-up with Infectious Disease to complete his isoniazid and rifampin therapy.

## 3. Discussion

The culprit of this case is BCG, a well-known subspecies of *M. bovis*. Infection following BCG administration is rare. In their review of tuberculosis (TB) cases in the United States from 2006 to 2013, Scott et al. found that of the 59,273 culture-positive cases, only 73 were linked to BCG [4].

While mainly used as a vaccine against TB, interest in the organism's antagonistic relationship with cancer has been documented since at least 1950 when studies began showing lower rates of leukemia in neonates immunized with BCG [5]. In 1976, Morales et al. described the antitumor effects of intravesical administration of BCG in eradicating carcinoma in nonmuscular invasive bladder cancer [5]. BCG immunotherapy has since become a first-line therapy for superficial bladder tumors and carcinomas in situ immediately after TURBT to reduce recurrence and progression of cancer [1]. While the mechanism of therapy is being elucidated, there is strong evidence for the role of the innate immune cells, CD4+ cells including Th1 cells, CD8+ cells, and granulocytes [5].

In the United States, bladder cancer accounts for 4.5% of cancers and 3% of cancer deaths [6]. In 2017, the prevalence was approximately 713,000 individuals [6]. However, rare efforts have been made to quantify the adverse outcomes of BCG immunotherapy in bladder cancer patients. A review of BCG immunotherapy in bladder cancer by Asín et al. found 282 instances of BCG-related complications between 1975 and 2013 [2]. The rate of complications was 5%, and the most frequently reported complication was disseminated BCG, found in 34% of the complicated cases. Other reported complications included allergic reaction, cystitis, hematuria, contracted bladder, ureteral obstruction, and, as seen in this case, vertebral disease. Another literature review found 18 cases of vertebral osteomyelitis from BCG making this report the 19^th^ documented case in the 40-year history of BCG immunotherapy for bladder cancer [7].

This case highlights the importance of having a high index of suspicion for BCG therapy-related etiology following TURBT, particularly in cases involving progressive lower back pain with hematuria. While hematuria has an incidence near 1%, in cases with repeated hematuria following subsequent BCG therapy, disseminated disease should be suspected and monitored closely. In cases where the adverse reactions are more severe, cessation of therapy should be considered using a model such as the one proposed by Asin et al. [2].

Prompt diagnosis and intervention can prevent progression of disease with initiation of anti-mycobacterials and remove the need for surgery. In this case, delay in the diagnosis initially led to treatment of a presumed L3-L4 fracture through neurosurgery which ultimately failed to improve symptoms. It was only after surgical biopsy that the true etiology could be determined.

Vertebral osteomyelitis is the defining feature of this case and is an exceedingly rare presentation of an already rare disease. Generally, the incidence of vertebral osteomyelitis is 2.5 per 100,000 population and is often secondary to pyogenic bacteria including *S. aureus*, *E. coli*, and coagulase-negative *Staphylococcus* [3]. *Mycobacterium*-induced spondylodiscitis is referred to as Pott's disease and is responsible for 46% of vertebral osteomyelitis in developing countries [3]. For this reason, mycobacterial etiology is strongly suspected for vertebral osteomyelitis in developing countries. While Pott's disease is associated with *M. tuberculosis*, BCG can also be responsible. BCG-derived Pott's disease symptoms can frequently be mistaken for spinal bone metastasis due to this disease being a consequence of bladder cancer therapy [2].

Prior literature has shown the demographics of BCG osteomyelitis secondary to bladder cancer therapy to be exclusively male patients between the ages of 58 and 94 with symptoms occurring between 2 weeks and 12 years following intervention [3, 8]. As seen in this case, the primary spinal involvement is in the thoracolumbar region, namely, T6–L5, with secondary symptoms including weight loss and lower extremity radiculopathy subsequent to fever and night sweats [3]. Our patient also experienced chronic, progressive lower back pain with more specific symptoms including hematuria and a psoas abscess [8, 9].

The proposed pathogenesis of Pott's disease and other disseminated conditions from BCG immunotherapy arises from injury to the bladder urothelium from the treatment itself, urinary tract infection, traumatic catheterization, or transurethral resection of the tumor [2, 3]. Specific risk factors associated with spondylodiscitis include breach of the bladder mucosa with specific associations following prostatectomy, transurethral stone removal, and nephroureterectomy [2]. Once the pathogen enters the bloodstream, it can seed hematogenously to the thoracolumbar spine by traveling from the vesical venous plexus, to the prostatic venous plexus, and then directly into the vertebral venous plexus [2, 3]. The importance of the prostatic plexus in vertebral osteomyelitis is supported by the exclusively male patient population despite the use of BCG immunotherapy in women at a similar frequency. Mackel et al. propose that the lack of corresponding structure requires a circuitous route through the internal iliac veins to reach the vertebral plexus which is why that particular complication is not seen in this population [3].

Diagnosis of Pott's disease or disseminated BCG in general can be challenging. Extrapulmonary findings of disseminated BCG are difficult to detect with sputum smears, with only 4 of 118 total cases reported to the CDC from 2004 to 2015 testing positive [10]. Emphasis must be placed on sampling multiple locations suspected of disease and using genetic assay such as spacer oligonucleotide, mycobacterial interspersed repeating units, and variable number of tandem repeats [4]. For Pott's disease specifically, PCR and DNA probes for BCG following diagnosis of spondylodiscitis should be part of the formal testing in patients with a history of BCG immunotherapy for bladder cancer [7].

Our patient presented with the common finding of severe back pain with radiculopathy and had early imaging that suggested either a degenerative or early infectious etiology. Biopsies taken showed granulomatous infection with negative staining. Two weeks later, he presented with symptoms similar to Pott's disease including fever, night sweats, and severe lower back pain. Repeat imaging indicated osteomyelitis, discitis, and psoas muscle abscesses. Except for granulomatous findings on biopsy, there were no specific signs to a mycobacterial etiology until five days after treatment. Our patient's rare diagnosis was based on the repeated imaging findings showing spondylodiscitis, nonspecific granulomatous biopsy findings, and the history of BCG treatments for bladder cancer, which was a key component in guiding clinical suspicion which was later confirmed using PCR analysis.

Currently, there are no guidelines for treatment of vertebral osteomyelitis, and every attempt should be made to treat without surgical intervention. This means using pathogen-directed treatments. Like *M. bovis*, BCG, is almost universally pyrazinamide resistant [4]. Therapy for disseminated TB consists of isoniazid with B6 supplementation, rifampin, and ethambutol for two months. If the bacteria are susceptible to isoniazid and rifampin at the end of this intensive phase, ethambutol can be discontinued, and the patient continues on the remaining medications for 9 months. In previous cases, follow-up varied but usually consisted of isoniazid and rifampin for 6–12 months [8]. Monitoring of the spinal lesion every month with repeat CT/MRI is crucial to assess spinal stability, neurological compromise, or focal deficit development [7]. According to Mackel et al., “…indications for surgical debridement, spinal decompression, and stabilization include the progression of infection despite antimicrobial therapy, spine deformity, pathologic fracture with intractable pain, and neurologic compromises, such as myelopathy and radiculopathy” [3]. Surgical options in patients who fail to fuse or demonstrate spinal instability include decompression, fusion, and supportive instrumentation [7]. Our patient had laminectomies performed by Neurosurgery, but the mainstay of his treatment is empiric anti-mycobacterial treatment as described above. Fortunately, following a subsequent spinal stabilization surgery, he was able to fully recover and has remained symptom free illustrating the effectiveness of this anti-mycobacterial treatment protocol with surgical intervention. We hope this case adds to the limited literature to help development of guidelines and medical management for what we believe could be an underreported complication of BCG therapy.

## Figures and Tables

**Figure 1:**
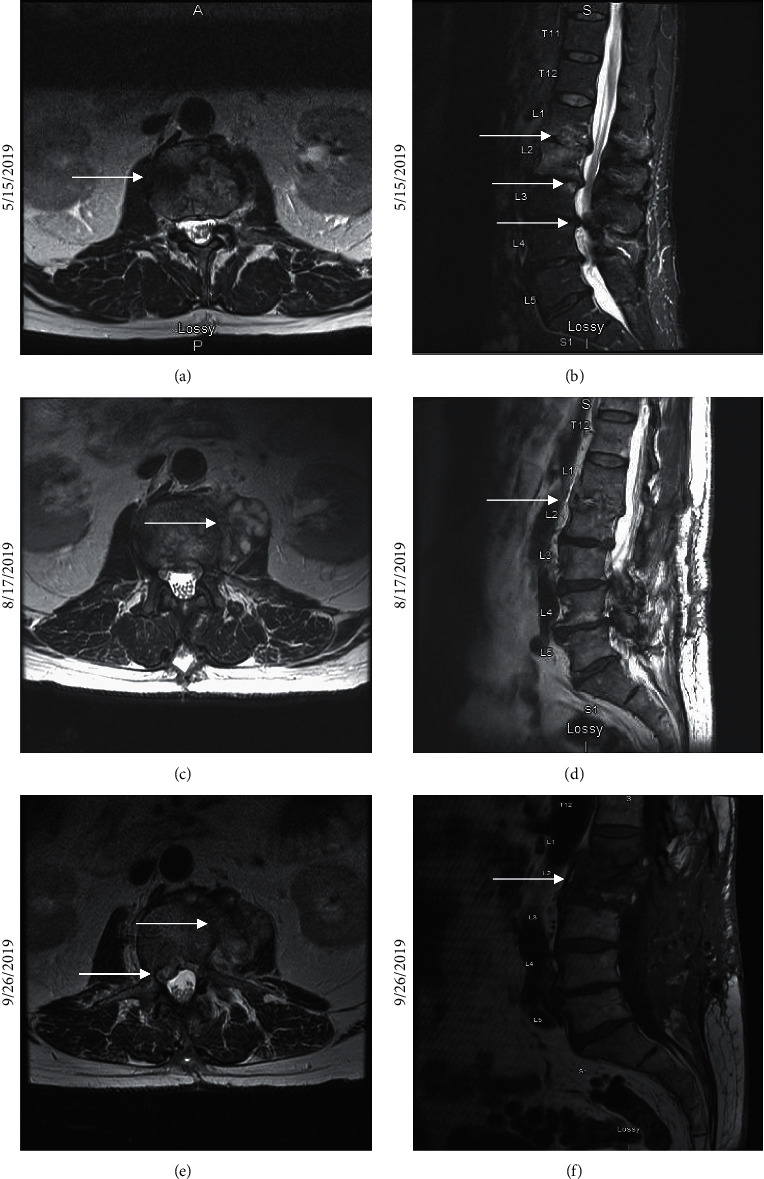
(a) An axial T2-weighted MRI image of morphological changes at the L1-L2 level presumed to be from degenerative process but difficult to distinguish from early infection. (b) A sagittal STIR MRI image showing multilevel degenerative changes resulting in severe canal stenosis particularly at the L2-L3 and L3-L4 levels. The morphological changes observed in Figure 1(a) are also visible. (c) An axial T2-weighted MRI image showing a heterogenous left paraspinal spinal mass with solid and cystic components. These findings raise concern for a potentially neoplastic process superimposed on an atypical infection. (d) A sagittal T1-FLAIR MRI image showing progressive, extensive degeneration at the L1-L2 and L3-L4 levels since prior study, suggesting infectious etiology. (e) An axial T1 FS post-MRI image showing 2 new small nonenhancing abscesses within the left psoas muscle and right L2-L3 paraspinal phlegmon. This epidural phlegmon, together with the microabscesses, caused an apparent impingement of the right L2 nerve at the right proximal margin of the right L2-L3 neural foramen. (f) A sagittal T1 MRI image showing L1-L2 discitis and vertebral, endplate osteomyelitis of L2.

## Data Availability

All previously used data can be found in the references.
